# Antibiotic resistance, ability to form biofilm and susceptibility to copper alloys of selected staphylococcal strains isolated from touch surfaces in Polish hospital wards

**DOI:** 10.1186/s13756-017-0240-x

**Published:** 2017-08-14

**Authors:** Anna Różańska, Agnieszka Chmielarczyk, Dorota Romaniszyn, Małgorzata Bulanda, Monika Walkowicz, Piotr Osuch, Tadeusz Knych

**Affiliations:** 1 Jagiellonian University Medical College, Faculty of Medicine, Chair of Microbiology, Czysta str. 18, 31-121 Kraków, Poland; 2 Faculty of Non-Ferrous Metals, Department of Metal Working and Physical Metallurgy of Non-Ferrous Metals, AGH University of Science and Technology, al. Mickiewicza 30, 30-059 Kraków, Poland

**Keywords:** Hospital environment contamination, Staphylococci, Antibiotic resistance, Antimicrobial copper, Biofilm-forming staphylococci

## Abstract

**Background:**

Despite the employment of sanitary regimes, contact transmission of the aetiological agents of hospital infections is still exceedingly common. The issue of microbe transmission becomes particularly important when facing multidrug-resistant microorganisms such as methicillin-resistant staphylococci. In the case of deficiencies in cleaning and disinfection procedures, hospital equipment made of copper alloys can play an important role, complementing traditional hospital hygiene procedures.

The objective of this study was to characterize staphylococcal strains isolated from touch surfaces in Polish hospital wards in terms of their drug resistance, ability to form biofilm and susceptibility to antimicrobial activity of copper alloys.

**Methods:**

The materials for the study were 95 staphylococcal strains isolated from touch surfaces in 13 different hospital wards from Małopolska province (the south of Poland). Phenotypic and genotypic antibiotic resistance were checked for erythromycin, clindamycin, gentamycin, ciprofloxacin, trimethoprim/sulfamethoxazole and mupirocin. Biofilm formation ability for the tested strains was checked with the use of culture on Congo red agar. Susceptibility to copper, tin bronze, brass and new silver was tested using a modification of the Japanese standard.

**Results:**

Over 67% of the analysed staphylococcal strains were methicillin-resistant (MR). Four strains were resistant to all of the tested antibiotics, and 14 were resistant to all except mupirocin. Strains classified as MR had significantly increased resistance to the remaining antibiotic groups. About one-third of the analysed strains revealed biofilm-forming ability. Among the majority of species, biofilm-forming and non-biofilm-forming strains were distributed evenly; in the case of *S. haemolyticus* only, negative strains accounted for 92.8%. Susceptibility to copper alloys was different between strains and rather lower than in the case of the SA strain selected for comparison.

**Conclusions:**

Coagulase-negative staphylococci, the most commonly isolated in Polish hospital wards, should not be neglected as an infection risk factor due their high antibiotic resistance. Our experiments confirmed that touch surfaces made of copper alloys may play an important role in eliminating bacteria from the hospital environment.

## Background

Despite the employment of sanitary regimes, contact transmission of the aetiological agents of hospital infections is still exceedingly common [1]. The issue of microbe transmission becomes particularly important when multidrug-resistant microorganisms, such as methicillin-resistant staphylococci (MRSA, MRCoNS), vancomycin-resistant enterococci (VRE), or non-fermentative bacilli, appear in the hospital environment [2]. The spread of methicillin-resistant *S. aureus* (MRSA) or *S. epidermidis* (MRSE) outside hospital units (hotels, buses) is also described, but it is of greater significance when the drug-resistant bacteria present themselves in hospitals [3–6]. Methicillin-resistant staphylococcal strains are frequently resistant not only to beta-lactam antibiotics but also to the majority of antibiotics used for therapy. Rapidly increasing resistance among both staphylococci and Gram-negative bacilli is a very serious problem in hospitals as it limits effective treatment of infections, especially in patients with severe infections treated in ICUs [7, 8]. The most common vector mediating the transfer of microbes is the hands of the staff and of patients, and the reservoir is represented by surfaces most often touched (door handles, countertops, light switches and others), on which these microorganisms can survive over long periods of time – many days or even months [9]. Cleaning and disinfection of these surfaces is an action obviously applied that has significance in controlling MRSA and MRCoNS [9]. Solutions to this problem are searched for in many directions, and understanding bacterial mechanisms of resistance enables one to seek new drugs tackling this resistance, but there is also a search for new substances that could be used in disinfectants and new materials (e.g., metal alloys) for use in commercial (touch) surfaces.

The objective of the present study was to describe the antibiotic resistance (phenotypes and genotypes), the ability to create biofilm and the susceptibility to selected copper alloys of the most numerous staphylococcal strains (*n* = 95) isolated from hospital unit touch surfaces.

## Methods

The materials for the study were strains cultured from touch surfaces in 13 different units in three hospitals of various sizes and profiles located in Małopolska province (the south of Poland). Swabs for testing were collected from the following surfaces that may be a reservoir for microbes: worktop in sickroom, bedside table top, drip stand, bed frame, soap dispenser, disinfecting fluid dispenser, light switch, ventilator monitor, mobile phone, department landline phone receiver, computer keyboard, dressing (or surgical) trolley worktop, door handle, protective glove container, and tissue package. The detailed methodology and results of environmental screening are described elsewhere [10], but the most numerous isolates were coagulase-negative staphylococci (85.7%), followed by *Staphylococcus aureus* (2.7%), streptococci (including *E. faecalis*) (8.9%), Gram-negative bacilli (1.8%) and others (0.9%). In the most numerous group of coagulase-negative staphylococci, the following species were isolated: *Staphylococcus epidermidis, Staphylococcus haemolyticus*, *Staphylococcus hominis*, *Staphylococcus warneri*, *Staphylococcus capitis*, *Staphylococcus simulans*, *Staphylococcus pettenkoferi*, *Staphylococcus caprae* and coagulase-positive ones - *S. aureus.*

The collected strains were stored in the Department of Microbiology at −70 °C (according to the procedure of Microorganism Preservation System Protect TS/80-MX).

### Drug resistance study

All isolates were tested using disk diffusion antimicrobial susceptibility methods on Mueller-Hinton agar plates according to the current guidelines of the European Committee on Antimicrobial Susceptibility Testing (EUCAST Tables v. 6.0; http://www.eucast.org v.6.0 accessed 1.12.2016). Antibiotics used in this study included erythromycin (2 μg), clindamycin (15 μg), gentamycin (10 μg), ciprofloxacin (5 μg), trimethoprim/sulfamethoxazole (1.25/23.75 μg), and mupirocin (200 μg). All disks were obtained from Oxoid (Basingstoke, United Kingdom). The MRSA resistance phenotype was detected using a cefoxitin disc (30 μg) according to the current EUCAST guidelines. The macrolide-lincosamide-streptogramin B (MLSB) resistance phenotype of the isolates was determined by D-test according to a previously published protocol [11].

### DNA isolation

DNA was extracted from isolates using the Genomic Mini kit (A&A Biotechnology, Gdynia, Poland) according to the manufacturer’s instructions.

### Polymerase chain reaction (PCR) screening for selected resistance genes

MRSA phenotype was confirmed by detection of the *mec*A gene in PCR amplification using previously published primers (Table 1) [12]. Erythromycin resistance genes (*erm*A, *erm*B, *erm*C, and *msr*A/B) were detected by multiplex PCR, and amplification of a 456 bp fragment of the *mup* gene (mupirocin resistance gene) was performed by single PCR (Table 1) [13, 14]. As positive controls, *S. aureus* ATCC 33591 and ATCC BAA 1708 were employed.

**Table 1:** Sequences of starters used for detection of resistance genes

Gen	Primer sequences 5′-3′	Product of amplification	References
*mecA*	TAG AAA TGA CTG AAC GTC CG TTG CGA TCA ATG TTA CCG TAG	154 bp	Pereira 2010 [12]
*mup*	TAT ATT ATG CGA TGG AAG GTT GG AAT AAA ATC AGC TGG AAA GTG TTG	458 bp	Anthony RM 1999 [14]
*ermA*	TCT AAA AAG CAT GTA AAA GAA CTT CGA TAG TTT ATT AAT ATT AGT	645 bp	Sutcliffe 1996 [13]
*ermB*	GAA AAG GTA CTC AAC CAA ATA AGT AAC GGT ACT TAA ATT GTT TAC	639 bp	Sutcliffe 1996 [13]
*ermC*	TCA AAA CAT AAT ATA GAT AAA GCT AAT ATT GTT TAA ATC GTC AAT	642 bp	Sutcliffe 1996 [13]
*msrA/B*	GCA AAT GGT GTA GGT AAG ACA ACT ATC ATG TGA TGT AAA CAA AAT	399 BP	Sutcliffe 1996 [13]

### Biofilm

**Fig. 1:**
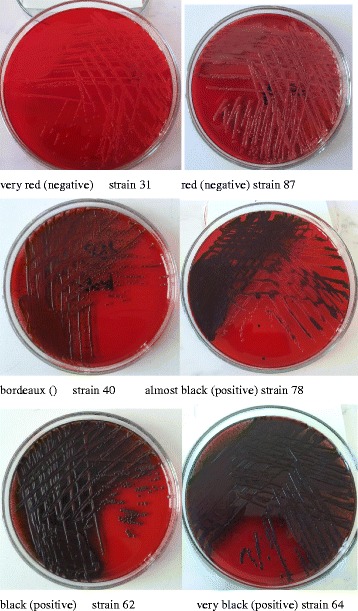
Examples of strains with and without ability to biofilm formation on plates with Congo *red* agar

Isolate biofilm formation was screened using the method described by Arciola et al. [15] with Congo red agar (CRA). CRA plates were prepared by adding 0.8 g of Congo red and 36 g of saccharose (Sigma, Missouri, USA) to 1 L of brain heart infusion agar (Oxoid, Basingstoke, Hampshire, England). The plates were incubated for 24 h at 37 °C and subsequently overnight at room temperature. On CRA, slime-producing strains form black colonies, whereas non-producing strains develop red colonies. For an accurate assessment of all the possible chromatic variations exhibited by the cultured colonies, a six-colour scale was used [15]. The scale ranged from very black (vb), through black (b), and almost black (ab) to bordeaux (brd, red (r) and very red (vr) (Fig. 1). Very black and black colonies were considered normal slime producer strains, while dark, almost black colours were considered indicative of weak slime production activity. Conversely, very red to bordeaux coloured colonies were considered grades of red and classified as strains unable to produce slime.

### Chosen copper alloys and their preparation

Metal samples measuring 2.5 cm × 2.5 cm with a thickness of 1–2.5 mm were provided by the Faculty of Non-ferrous Metals, AGH University of Science and Technology, Kraków. Before their delivery for microbiological testing, the samples underwent mechanical polishing, cleaning and degreasing by immersion in acetone. Prior to use for microbiological testing, the samples were sterilized by wiping with 96% alcohol. Studies were conducted on the following copper alloys: brass CuZn37, tin bronze CuSn6, and nickel silver CuNi18Zn20 with ETP copper (99.9% Cu) as a positive control (presumed highest antimicrobial efficacy) and stainless steel as a negative control (assumed lack of antimicrobial properties). The copper alloys selected for this study are the most well known and most frequently used in various industries. The alloys used in the study with data on the concentration percentage of copper are listed in Table 2.

**Table 2:** Compositions (%) of the tested commercial copper alloys

Common name	UNS code	Cu	As	Bi	Cd	Fe	Mn	Al	Ni	P	Pb	Sb	Si	Sn	Zn
Copper Cu-ETP	C11000	99.9	0.0	0.001	0.001	0.002	0.001	0.0	0.0	0.030	0.002	0.000	0.008	0.0	0.0
Yellow Brass CuZn37	C27400	63.2	0.001	0.001	0.001	0.001	0.001	0.001	0.06	0.001	0.004	0.001	0.008	0.0	36.7
Phosphor Bronze CuSn6	C51900	94.1	0.006	0.002	0.0	0.001	0.001	0.016	0.01	0.222	0.038	0.001	0.002	5.5.	0.1
Nickel silver CuNi18Zn20	C75200	63.1	0.001	0.001	0.001	0.027	0.12	0.001	17.9.	0.001	0.001	0.008	0.001	0.001	18.9.
Stainless Steel	S30400	Fe 68.8, C 0.07, Cr 19, Mn 2, N 0.1, Ni 10, P 0.045, S 0.015, Si 1													

*UNS* (Unified Numbering System), *ETP* Electrolytic Tough Pitch

### Quantitative culture method to determine the antimicrobial effectiveness of copper and its alloys

In this study, wet exposure was used (modified methodology of the Japanese Standard [16]) to assess the antimicrobial properties of the selected copper alloys. The testing procedure is described in detail below.

For susceptibility to copper alloys four CNS strains were chosen and, for comparison, one *Staphylococcus aureus* strain (ATCC 12493). The following strains were chosen to examine antimicrobial properties of copper alloys: *S. epidermidis* no. 65 (MSSE), also sensitive to ciprofloxacin and gentamicin, biofilm-forming (category: black); *S. epidermidis* no. 62 (MRSE), resistant to the remaining antibiotics, biofilm-forming (category: black); *S. haemolyticus* no. 93 (MSSH), sensitive to all antibiotics, non-biofilm-forming (category: bordeaux) and *S. haemolyticus* no. 89 (MRSH), resistant to the remaining antibiotics, non-biofilm-forming (category: bordeaux) (Table 3). The strain numbers are internal laboratory numbers in this study.

**Table 3:** Characteristic of selected for tests of susceptibility for copper alloys CNS strains

Number of strain/strain code	Species	Biofilm	Cefoxitin resistance
65/ MSSE 65	*S.epidermidis*	black (+)	S
62/ MRSE 62	*S.epidermidis*	black (+)	R
93/ MSSH 93	*S.haemolyticus*	bordeaux (−)	S
89/MRSH 89	*S.haemolyticus*	bordeaux (−)	R
ATCC 12493	*S. aureus*	very black (+)	R

The tested bacterial strains were stored in glycerol at −70 °C. One day before antimicrobial efficacy testing, a small amount of the suspension was taken from a frozen sample, inoculated onto solid Muller-Hinton agar (MHA, BIOCORP, Warsaw, Poland) (clean culture) and then incubated for 24 h at 37 °C. From the obtained culture, a suspension was prepared in saline at a density of 0.5 McFarland standard (controlled using a densitometer bioSan, Ryga, Latvia). Subsequently, 100 μL of the suspension with a density of 0.5 McFarland standard was transferred to 900 μL of TSB. Each time, a control for the viability of the bacteria obtained in the culture on solid medium and a control for the precise initial concentration (its density expressed in CFU/mL) was performed.

Samples of the metals tested were placed in a sterile container made of PVC with a capacity of 100 mL that was 6 cm in diameter, and then, 100 μL of a test suspension was applied (the composition depended on the variant of the experiment). Next, the container was covered with sterile polypropylene foil measuring 2 cm × 2 cm to provide close contact between the bacterial suspension and the metal surface. The container was covered to prevent contamination of the sample with microbes from the air, but it remained loose enough that aerobic conditions were maintained throughout the course of exposure and when left for a specified period of time (0, 30, 60, 90, 120, 180, 240, and 300 min) at approx. 22 °C (room temperature).

After a certain period of time, 5 mL of TSB solution and approx. 30 sterile glass beads that were 2 mm in diameter were placed into the container and shaken for 2 min in a shaker (shaker-incubator ES-20/60, Ryga, Latvia). Then, 100 μL of the wash was collected, and 4 serial decimal dilutions were prepared, of which 100 μL was inoculated onto solid MHA for each time-point. After a 24-h incubation, individual colonies were counted on the plates when the resulting number was countable.

For each metallic material, each exposure time for all microbes was repeated three times. To count the amount of CFU/mL after exposure of the bacterial suspension to the studied materials, the average of triplicates was used. The formula for the calculation was CFU/mL = (n x f x v1)/(v2 x v3), where n is the average number of colonies/plate in dilution, f is the dilution factor, v1 is the volume of TSB used for washing the bacteria that survived after exposure, v2 is the volume used and applied on metallic coupons, and v3 is the volume of the plated material (v1-3 in mL).

**Fig. 2:**
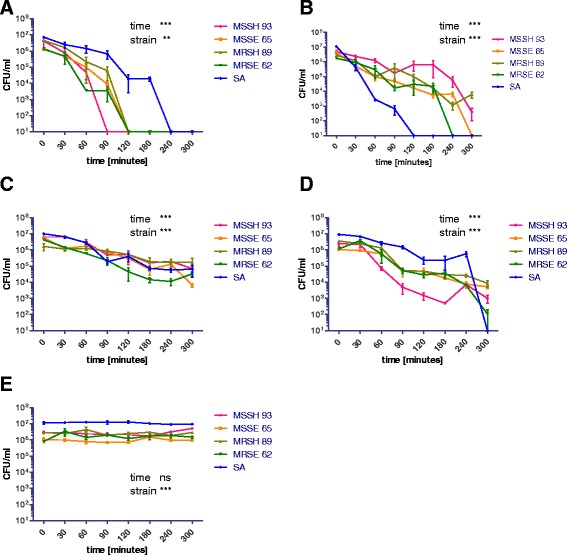
Tested bacteria inoculum density (CFU/mL) reduction on metallic materials in chosen time periods. **a** for Cu-ETP, **b** for CuSn6, **c** for CuZn37, **d** for CuNi18Zn20, **e** for stainless steel. MSSH 93 - methicillin sensitive *Staphylococcus haemolyticus*, strain no. 93, MSSE 65 - methicillin sensitive *Staphylococcus epidermidis*, strain no. 65, MRSH 89 - methicillin-resistant *Staphylococcus haemolyticus*, strain no. 89, MRSE 62 - methicillin-resistant *Staphylococcus epidermidis* strain no. 62, SA – *Staphylococcus aureus* ATCC 12493. ^***^ - *p* < 0.001, ^**^ - *p* < 0.01, ns – not statistically significant

To evaluate the effectiveness of the antimicrobial activity, the criteria used by Souli et al. [17] were adopted, according to which a suspension density reduction ranging from ≤2 to <3 log means the presence of bacteriostatic properties, whereas a reduction of >3 log means bactericidal properties. Ole et al. estimated that the microbiological load of touch surfaces, such as door handles in hospital wards, was 1–6 × 10^3^ CFU [18] and therefore, the criteria proposed by Souli et al. can be deemed to be appropriate for testing the antimicrobial efficacy of products made of copper and its alloys. The results are presented in Fig. 2.

### Statistical analysis

Test of comparison between fractions was used for analysis of statistical significance of antimicrobial resistance within CoNS species and between strains presenting resistance vs. susceptibility to methicillin – in case of characterization of antimicrobial resistance.

Results of susceptibility tests for the copper alloys tested were shown as charts of CFU/ml values in chosen time periods and were expressed as the mean ± SEM. Two-way Anova with repeated measures analysis of variance was used to evaluate the effects of both time and strain for every tested alloy. *P* values less than 0.05 were considered statistically significant.

## Results

Microbes of the genus *Staphylococcus* accounted for the vast majority of the population of bacteria cultured from touch surfaces in the hospital environment. Among staphylococci, of which almost 97% were CoNS *(coagulase-negative staphylococci)*, there were 8 different species, the most frequent of which were *S. epidermidis, S. haemolyticus* and *S. hominis*, accounting for 34%, 30% and 15% of all CNS isolates, respectively.

### Drug resistance

More than 67% (*n* = 64) of the analysed staphylococcal strains were methicillin-resistant, which is equivalent to resistance to all beta-lactam antibiotics. The presence of the *mec*A gene was confirmed in 68.4% of isolates (*n* = 65). Two *S. haemolyticus* isolates that were resistant to cefoxitin did not have the *mec*A gene, and three *S. epidermidis* isolates had the *mec*A gene and simultaneous susceptibility to cefoxitin (Table 4).

**Table 4:** Distribution of phenotypic and genotypic antibiotic resistance among tested staphylococcal strains

Species	n (%)	Methicillin resistance phenotype n (%)	Gen mecA n (%)	Phenotypes of macrolides resistance			erm (A, B, C) genes n (%)	Msr gen n (%)	Mupirocin resistence - phenotype n (%)	Mup gen n (%)
				cMLSB n (%)	iMLSB n (%)	MSB n (%)				
*S. epidermidis*	32 (33.7)	20 (21.1)	23 (24.2)	9 (9.5)	5 (5.3)	8 (8.4)	1 (1.1)	10 (10.5)	8 (8.4)	7 (7.4)
*S. haemolyticus*	28 (29.5)	22 (23.2)	20 (21.1)	17 (17.9)	0 (0)	5 (5.3)	0 (0)	15 (15.8)	3 (3.2)	6 (6.3)
*S. hominis*	14 (14.7)	13 (13.7)	13 (13.7)	3 (3.2)	6 (6.3)	2 (2.1)	0 (0)	0 (0)	2 (2.1)	5 (5.3)
*S. capitis*	6 (6.3)	4 (4.2)	4 (4.2)	3 (3.2)	0 (0)	0 (0)	0 (0)	1 (1.1)	1 (1.1)	0 (0)
*S. warnerii*	6 (6.3)	1 (1.1)	1(1.1)	0 (0)	0 (0)	2 (2.1)	0 (0)	2 (2.1)	0 (0)	0 (0)
*S. simulans*	3 (3.2)	1 (1.1)	1 (1.1)	0 (0)	3 (3.2)	0 (0)	0 (0)	0 (0)	0 (0)	0 (0)
*S. aureus*	3 (3.2)	1 (1.1)	1 (1.1)	1 (1.1)	0 (0)	0 (0)	0 (0)	0 (0)	0 (0)	0 (0)
*S. pettenkoferi*	2 (2.1)	2 (2.1)	2 (2.1)	1 (1.1)	1 (1.1)	0 (0)	0 (0)	0 (0)	0 (0)	0 (0)
*S. caprae*	1 (1.1)	0 (0)	0 (0)	0 (0)	0 (0)	0 (0)	0 (0)	0 (0)	0 (0)	0 (0)
Total	95 (100)	64 (67.4)	65 (68.4)	34 (35.8)	15 (15.8)	17 (17.9)	1 (1.1)	28 (29.5)	14 (14.7)	18 (18.9)

*n* number

In the group of the remaining antibiotics, the highest level of resistance was to erythromycin (70.5%) and clindamycin (52.6%). Resistance to gentamycin was demonstrated by 43.2% of the isolates. Resistance not exceeding 40% was found to ciprofloxacin, SXT and mupirocin (Table 4). Four strains were resistant to all of the tested antibiotics, and 14 to all except for mupirocin.

Resistance to macrolides and lincosamides can be conditioned by three mechanisms. The first mechanism, consisting of modification of the destination on the ribosome, was detected in 51.6% (*n* = 49) of the isolates (constitutive resistance phenotype cMLSB and inducible iMLSB). Expression of cMLSB resistance occurred in 34 isolates (35.8%) and that of iMLSB in 15 isolates (15.8%). The gene encoding the ribosomal methylase enzyme (*erm*B) was detected in only one strain from that group, and the *msr*A gene encoding the membrane transporter was detected in 15 isolates from that group.

The second mechanism of resistance is active removal of the antibiotic from the cell (efflux). The phenotype of resistance to erythromycin and preserved susceptibility to clindamycin (MSB) was confirmed in 17 isolates (17.9%), and the *msr*A gene was detected in 12 isolates from this group. One isolate had the *msr*A gene despite a lack of resistance to macrolides and lincosamides. The *msr* gene was thus detected in 28 isolates (29.5%).

Phenotypic resistance to mupirocin was found in 14 strains, and the *mup* gene was shown in 18 strains.

The incidence of MLSB and MSB phenotypes correlated with the incidence of MRCoNS and MRSA phenotypes; 52 (54.8%) isolates had both of these mechanisms at the same time.

Strains classified as MRCoNS had significantly increased resistance to the remaining antibiotic groups (*p* < 0.00001) (Table 5), but no statistically significant differences were observed in the proportion of methicillin resistance in specific CoNS species (*p* = 0.297).

**Table 5:** Antibiotic resistance of methicilline-resistant vs. sensitive staphylococcal strains isolated from touch surfaces

Antibiotic tested	Resistance for other antibiotics of CoNS (total) (n, %)	Resistance for other antibiotics of MRCoNS strains (n, %)	Resistance for other antibiotics of MSCoNS strains (n, %)
cefoxitin	63 (66.3)	63 (66.3)	29 (30.5)
erythromycin	66 (69.5)	53 (55.8)	13 (13.7)
clindamycin	49 (51.6)	43 (45.3)	6 (6.3)
ciprofloxacin	37 (38.9)	35 (36.8)	2 (2.1)
gentamycin	40 (42.1)	35 (36.8)	5 (5.3)
trimethoprim/sulfamethoxazole	33 (34.7)	31 (32.6)	2 (2.1)
mupirocin	14 (14.7)	12 (12.6)	2 (2.1)

*MR* methicillin resistant, *MS* methicillin sensitive

### Examination of antimicrobial activity of copper alloys on selected CoNS vs. SA strains

The material with greatest bactericidal properties is ETP copper, with which the density of the initial suspension, when in contact with the copper, decreased from 10^6^ to 10^7^ CFU/mL depending on the strain, and reached zero density in less than 4 h. For three of the tested CoNS strains, a complete reduction was observed after 2 h (Table 6). Three out of four tested coagulase-negative staphylococcal strains underwent complete reduction quicker than the SA strain, whereas the time needed for complete reduction of the MSSH93 strain was the same as for SA (Table 6).

**Table 6:** Tested bacteria inoculum density (CFU/mL) reduction on Cu-ETP, CuSn6, CuZn37, CuNi18Zn20, stainless steel, in chosen time periods

Time [min]	MSSE 65^a^	MRSE 62	MSSH 93	MRSH 89	SA
Cu-ETP					
0	1.25E + 06	1.38E + 06	4.22E + 06	4.52E + 06	7.49E + 06
30	4.83E + 05	4.67E + 05	7.50E + 05	1.68E + 06	2.33E + 06
60	1.00E + 05	3.50E + 03	3.33E + 04	8.60E + 05	7.92E + 05
90	8.50E + 03	4.17E + 03	1.67E + 05	3.73E + 04	3.87E + 05
120	1.00E + 00	1.00E + 00	8.33E + 05	1.00E + 00	4.67E + 04
180	1.00E + 00	1.00E + 00	1.67E + 05	1.00E + 00	1.00E + 04
240	1.00E + 00	1.00E + 00	1.00E + 00	1.00E + 00	1.00E + 00
300	1.00E + 00	1.00E + 00	1.00E + 00	1.00E + 00	1.00E + 00
CuSn6					
Time [min]	MSSE 65	MRSE 62	MSSH 93	MRSH 89	SA
0	4.43E + 06	1.73E + 06	5.05E + 06	2.68E + 06	1.13E + 07
30	5.17E + 05	9.00E + 05	2.35E + 06	1.23E + 06	4.33E + 05
60	1.00E + 05	3.00E + 05	1.23E + 06	1.32E + 05	3.08E + 03
90	4.50E + 04	1.67E + 04	1.72E + 05	3.90E + 05	1.17E + 03
120	1.50E + 04	3.00E + 04	2.48E + 05	9.67E + 04	1.00E + 00
180	5.00E + 03	2.17E + 04	6.37E + 05	1.17E + 04	1.00E + 00
240	1.67E + 03	1.00E + 00	5.83E + 04	1.67E + 03	1.00E + 00
300	1.00E + 00	1.00E + 00	1.00E + 00	1.10E + 05	1.00E + 00
CuZn37					
Time [min]	MSSE 65	MRSE 62	MSSH 93	MRSH 89	SA
0	5.62E + 06	4.17E + 06	6.17E + 06	1.68E + 06	1.01E + 07
30	1.25E + 06	1.43E + 06	6.38E + 06	1.13E + 06	6.55E + 06
60	1.72E + 06	5.83E + 05	2.83E + 06	1.25E + 06	2.78E + 06
90	7.33E + 05	6.67E + 04	3.67E + 05	8.50E + 05	1.47E + 05
120	3.02E + 05	4.00E + 04	5.00E + 05	5.25E + 05	3.97E + 05
180	6.33E + 04	1.67E + 04	1.83E + 05	1.88E + 05	7.10E + 05
240	1.30E + 05	8.33E + 03	1.92E + 05	1.68E + 05	4.70E + 05
300	6.67E + 03	3.33E + 04	3.17E + 04	1.72E + 05	6.50E + 04
CuNi18Zn20					
Time [min]	MSSE 65	MRSE 62	MSSH 93	MRSH 89	SA
0	1.05E + 06	1.17E + 06	2.45E + 06	4.25E + 05	9.20E + 06
30	9.17E + 05	3.68E + 06	2.18E + 06	2.25E + 05	6.77E + 06
60	5.67E + 05	5.67E + 05	1.23E + 05	3.63E + 05	2.70E + 06
90	4.83E + 04	3.67E + 04	5.00E + 03	5.33E + 04	1.50E + 06
120	4.50E + 04	3.00E + 04	1.00E + 00	3.52E + 05	2.38E + 05
180	2.00E + 04	3.67E + 04	1.00E + 00	1.83E + 04	2.12E + 05
240	5.00E + 03	8.33E + 03	1.00E + 00	1.67E + 04	5.77E + 05
300	1.67E + 03	1.00E + 00	1.00E + 00	2.50E + 04	2.83E + 03
SS					
Time [min]	MSSE 65	MRSE 62	MSSH 93	MRSH 89	SA
0	1.05E + 06	8.17E + 06	2.98E + 06	3.33E + 06	1.18E + 07
30	9.83E + 05	3.40E + 06	2.87E + 06	2.20E + 06	1.20E + 07
60	8.00E + 05	1.43E + 06	2.45E + 06	4.63E + 06	1.27E + 07
90	7.00E + 05	2.02E + 06	2.22E + 06	1.80E + 06	1.25E + 07
120	7.33E + 05	1.23E + 06	2.12E + 06	2.10E + 06	1.30E + 07
180	1.55E + 06	1.80E + 06	1.73E + 06	3.43E + 06	1.05E + 07
240	9.67E + 05	1.92E + 06	3.20E + 06	1.50E + 06	9.33E + 06
300	9.50E + 05	1.50E + 06	5.15E + 06	3.08E + 06	9.77E + 06

^a^strains codes – see Table 3

Slightly lower antibacterial efficacy was exhibited by tin bronze (CuSn6). In only one of the strains tested (resistant to all antibiotics investigated) was complete reduction not observed (MRSH89 strain) after 300 min of exposure, and there was even no evidence of bacteriostatic activity – the suspension density decreased over a period of 6 h from 2.68E + 06 to 1.10E + 05 (Table 6). When the tested strains were exposed to CuSn6, the fastest complete reduction in suspension density was demonstrated for SA – after 2 h; and for the studied CoNS strains, the fastest complete reduction was found for strain MRSE62, demonstrating biofilm-forming properties (Table 6).

With respect to all of the studied staphylococcal strains, brass (CuZn37) demonstrated bacteriostatic properties after 300 min; the reduction in suspension density ranged from ≥2 log <3 log (Table 6).

In the case of material made from CuNi18Zn20 (nickel silver), a complete reduction in suspension density was observed for one of the studied MRSE62 strains, resistant to the majority of the examined antibiotics and exhibiting biofilm-forming properties (Table 6). Regarding the MRSH89 strain, resistant to all of the tested antibiotics, the CuNi18Zn20 material displayed neither bactericidal nor bacteriostatic effects; the degree of reduction of the initial bacterial suspension for the remaining CoNS and SA strains was within the limits of bacteriostatic activity.

As for stainless steel, no bacteriostatic or bactericidal properties were found for any of the tested strains (Table 6).

The speed and degree of reduction of bacterial suspensions density for the tested strains on ETP copper, copper alloys and stainless steel, together with the results of statistical significance for strains and time points, are presented in Fig. 2. Only for stainless steel no significant reduction of bacterial density in time was observed. Significant differences were observed between strains for all tested metals (for stainless steel it was the result of difference in bacterial suspension density in time zero between SA and CoNS strains).

### Biofilm

The environmental strains subjected to analysis were examined in terms of their biofilm formation capabilities: there were 32 (33.7%) biofilm-forming strains (categories: very black, black and almost black), including very black – 1 strain, black – 11 and almost black – 20, and 63 (66.3%) non-biofilm-forming strains (categories: bordeaux, red and very red), including bordeaux – 54, red – 4, and very red – 5 (Fig. 1).

Among the majority of species, biofilm-forming and non-biofilm-forming strains were distributed evenly; in the case of *S. haemolyticus* only, negative strains accounted for 92.8%.

## Discussion

The staphylococcal strains that were found on touch surfaces were primarily CoNS. Fewer than 3% of *S. aureus* species found were coagulase-positive isolates*.* Staphylococcal strains on touch surfaces can come from both the normal human flora (hand skin) and the environment, especially the air. CoNS are not dangerous to people with a properly functioning immune system but, in hospitals, they may pose a threat especially for patients who are severely ill, older, immunocompromised, hospitalized in the ICU or newborns in the NICU [19, 20]. *S. epidermidis* *, S. haemolyticus,* and *S. hominis* are the most frequent CoNS found to be responsible for causing skin infections as well as severe invasive infections [21–23]. Additionally, treating infections caused by CoNS can be difficult due to their drug resistance [23, 24].

In our study, among CoNS species, *S. epidermidis* and *S. haemolyticus* were predominant, which differs from studies on air also conducted in the south of Poland where *S. saprophyticus* and *S. warneri* were prevailing [25]. The reason for this difference might be the different places of isolation (touch surfaces, not air) as well as the method applied for species identification. In this work, we employed the state-of-the-art and most recommended method, that is, Matrix Assisted Laser Desorption Ionization Time of Flight (MALDI-TOF). In other studies in which authors employed the MALDI method for environmental research, strains from the species *S. haemolyticus, S. hominis* [4], *S. haemolyticus* and *S. epidermidis* [26] were most common. Additionally, Mkrtchyan et al. showed that results may vary when different identification methods are applied [27].

*S. aureus*, which is the main cause of hospital-acquired infections, was not isolated in large numbers, which can be surprising but has been confirmed by recent studies in Poland and abroad [4, 25, 28].

Many CoNS strains were methicillin-resistant (as many as 65%), which greatly limits therapeutic options in the case of potential opportunistic infections that they cause, due to inapplicability of the entire group of beta-lactam antibiotics [29]. For the vast majority of isolates, phenotypic resistance was confirmed through the detection of the *mec*A gene. Three *S. epidermidis* strains had the *mec*A gene and retained sensitivity to cefoxitin – similar strains were found for the species *S. aureus* [30].

Surprisingly, many isolates were also resistant to erythromycin (over 2/3) and clindamycin (a 1/2). Additionally, almost 55% of isolates had simultaneous resistance to methicillin and the MLSB mechanism of resistance.

The most frequent phenotypically detected mechanism of resistance to macrolide antibiotics, lincosamides and streptogramins B was the constitutive MLSB mechanism (35.8%), which agrees with the study by Lina [31] (here, it was 36.6%) and is a little less than Castro-Alarcon’s results [32] (47%) but different from Lenart-Boroń’s findings (where it was present only in 4% of the strains and where the inducible mechanism was predominant) [25]. The phenotype of resistance to erythromycin and retained sensitivity to clindamycin (MSB) was confirmed in 17 isolates (17.9%), and for Lenart-Boroń, it was 28% [25]. The gene associated with resistance to macrolides that was most often detected in our study was the *msr*A gene encoding a pump responsible for active efflux of the antibiotic from the cell (29.5% of the strains); the *erm*B gene, one of the genes encoding a methylase, was present in only 1 strain. Castro-Alarcon had contrasting results in which 32% of strains had the *erm*A gene [32]. The differences between the genes detected and the phenotypic detection of mechanisms of resistance may stem from testing only several selected genes – we did not look, e.g., at different variants of the *msr* gene, *mph* genes (encoding phosphotransferase), or the flax gene (responsible for resistance to lincosamides). We also did not examine the problem of gene expression. Our aim was not only to study the mechanisms of resistance; we also wanted to select strains for research on antibacterial properties of copper alloys.

Sensitivity to mupirocin concerned the greatest number of strains, and the *mup* gene was found in approx. 18% of strains.

One of the significant virulence factors of CoNS strains is their ability to form biofilm. The biofilm testing method applied here is a screening method. However, it allowed us to estimate that approximately 1/3 of the strains are potentially biofilm-forming, fewer than in the study by Szczuka, where 64% of CoNS strains formed biofilm [33].

The copper alloys that we tested have Environmental Protection Agency (USA) certificates, but EPA recommendations are based on tests for such bacterial species as *Staphylococcus aureus*, *Escherichia coli*, *Enterobacter aerogenes* and *Pseudomonas aeruginosa*, which are the bacteria that cause the majority of hospital-acquired infections [34, 35]. Coagulase-negative staphylococci, however, have also been identified as aetiologic agents of infection [12, 15, 19], and their drug resistance and ability to form biofilms, demonstrated in this study and by other researchers [24, 26], justify activity assays of copper alloys for potential use on touch surfaces in hospital departments. Four strains of the most often isolated CoNS species were selected for research on their antimicrobial properties. They differed in their resistance to antibiotics and their ability to form biofilm, and our experiments also revealed differences in susceptibility to the copper alloys.

The tests conducted showed that only for Cu-ETP was similar or higher bactericidal activity found against the studied CoNS strains than against *Staphylococcus aureus* – the maximum bacterial suspension density reduction from the level of approx. 10^7^ CFU/mL to 0 was observed in 120 to 240 min, depending on the strain.

Tin bronze demonstrated a faster bactericidal effect against the tested SA strain. Complete reduction in suspension density was observed after 120 min, but in the case of three of the tested CoNS strains, this time was 240 or 300 min, while for the MRSH89 strain (methicillin-resistant, non-biofilm-forming), after 300 min, the reduction in suspension density was lower by 2 log.

A similar course and degree of reduction in initial suspension density was observed for all of the examined CoNS and SA on brass CuZn37 – the boundary criterion for determining bacteriostatic effects. On CuNi18Zn20, two of the tested CoNS strains demonstrated greater susceptibility than SA.

Noyce et al. examined sensitivity to copper and bronze for three different methicillin-resistant *Staphylococcus aureus* strains [36]. The authors observed similar susceptibility of the tested MRSA strains to copper (C19700) – total reduction of the initial suspension density from approx. 1E + 07 to zero in under 90 min – and for two MRSA strains on bronze (C24000), they found total reduction after approximately 4.5 h, while for one of the tested strains, the degree of reduction after 360 min did not meet the qualification criterion for bacteriostatic properties [36].

The study that we conducted shows that coagulase-negative staphylococci, most often isolated from touch surfaces in Polish hospital wards [13], are not only highly drug-resistant but also perhaps less sensitive to copper alloys than *Staphylococcus aureus*. However, most of the CoNS strains tested in this study demonstrated susceptibility to copper alloys, which as a result are useful as a complementary solution for traditional cleaning and disinfection in hospitals.

Additionally, in Poland, there are no official recommendations for testing the antimicrobial effectiveness of materials dedicated for touch surfaces in healthcare settings. In the case of developing such recommendations, perhaps research on antimicrobial properties of materials intended for use as touch surfaces should also include representative coagulase-negative staphylococci because, as was shown in this study, they can be a reservoir of antibiotic-resistance genes. The advantage of copper as a material with antimicrobial properties intended for touch surfaces is its cidal activity, consisting of the destruction of genetic material as shown in other studies [37], and hence the possibility that it can eliminate genes for antibiotic resistance.

## Conclusions

1. Coagulase-negative staphylococci, the most commonly isolated in Polish hospital wards, should not be neglected as infection risk factors due to their high antibiotic resistance.
2. About one-third of the tested environmental staphylococcal strains revealed biofilm-forming ability, and this may be a reason for the common contamination of hospital environments by these bacteria.
3. In the case of tin bronze, we observed lower antimicrobial activity against tested CoNS strains compared to an SA strain (longer times of bacterial density reduction). Despite this, our experiments confirmed that touch surfaces made of copper alloys may play an important role in eliminating bacteria from the hospital environment.

## Abbreviations

ATCC: American Type Culture Collection
CFU: Colony forming units
cMLSB: Constitutive macrolide-lincosamide-streptogramin B
CoNS: Coagulase-negative staphylococci
CRA: Congo red agar
EPA: Environmental Protection Agency
ETP: Electrolytic Tough Pitch
EUCAST: The European Committee on Antimicrobial Susceptibility Testing
iMLSB: Inducible macrolide-lincosamide-streptogramin B
MRCoNS: Methicillin-resistant coagulase negative staphylococci
MRSA: Methicillin-resistant *Staphylococcus aureus*
MRSE: Methicillin-resistant *Staphylococcus epidermidis*
MRSH: Methicillin-resistant *Staphylococcus haemolyticus*
MSB: Macrolide-streptogramin B
MSCoNS: Methicillin sensitive coagulase-negative staphylococci
MSSE: Methicillin sensitive *Staphylococcus epidermidis*
MSSH: Methicillin sensitive *Staphylococcus haemolyticus*
PCR: Polymerase chain reaction
R: Resistant
S: Sensitive
SA: *Staphylococcus aureus*
TSB: Tryptic soy broth
VRE: Vancomycin-resistant enterococci
